# Gene Expression Profiling of Dendritic Cells Reveals Important Mechanisms Associated with Predisposition to *Staphylococcus* Infections

**DOI:** 10.1371/journal.pone.0022147

**Published:** 2011-08-12

**Authors:** Mehdi Toufeer, Cécile M. D. Bonnefont, Eliane Foulon, Cécile Caubet, Christian Tasca, Marie-Rose Aurel, Christèle Robert-Granié, Rachel Rupp, Gilles Foucras

**Affiliations:** 1 Université de Toulouse, INP, ENVT; UMR 1225, IHAP, Toulouse, France; 2 INRA, UMR1225, IHAP, Toulouse, France; 3 INRA, UR631, SAGA, Castanet-Tolosan, France; 4 INRA, UE321, Roquefort, France; Statens Serum Institute, Denmark

## Abstract

**Background:**

*Staphylococcus aureus* is a major pathogen of humans and animals and emerging antibiotic-resistant strains have further increased the concern of this health issue. Host genetics influence susceptibility to *S*. *aureus* infections, and the genes determining the outcome of infections should be identified to find alternative therapies to treatment with antibiotics. Here, we used outbred animals from a divergent selection based on susceptibility towards *Staphylococcus* infection to explore host immunogenetics.

**Methodology/Principal Findings:**

We investigated how dendritic cells respond to heat-inactivated *S. aureus* and whether dendritic cells from animals showing different degrees of susceptibility had distinct gene expression profiles. We measured gene expression levels of *in vitro S. aureus*-stimulated bone marrow-derived dendritic cells at three different time points (0, 3 and 8 hrs) by using 15 k ovine Agilent microarrays. Furthermore, differential expression of a selected number of genes was confirmed by RT-qPCR. Gene signatures of stimulated DCs were obtained and showed that genes involved in the inflammatory process and T helper cell polarization were highly up-regulated upon stimulation. Moreover, a set of 204 genes were statistically differentially expressed between susceptible and resistant animals, and grouped them according to their predisposition to staphylococcal infection. Interestingly, over-expression of the *C1q* and *Ido1* genes was observed in the resistant line and suggested a role of classical pathway of complement and early regulation of inflammation pathways, respectively. On the contrary, over expression of genes involved in the IL1R pathway was observed in susceptible animals. Furthermore, the leucocyte extravasation pathway was also found to be dominant in the susceptible line.

**Conclusion/Significance:**

We successfully obtained *Staphylococcus aureus* associated gene expression of ovine BM-DC in an 8-hour kinetics experiment. The distinct transcriptional profiles of dendritic cells obtained from resistant and susceptible animals may explain susceptibility towards *S. aureus* infections in a broader context.

## Introduction

*Staphylococcus aureus* is a common bacterium causing diseases in a wide range of host species [1]–[4]. In contrast to its commensal status, *S. aureus* is also a very frequent pathogen causing infections of the skin and soft tissues, and sepsis. In humans and animals, it is a problem further compounded by the recent appearance of strains with broad antibiotic resistance, including methicillin-resistant *S. aureus* strains (MRSA) [5]. The duality of *S. aureus* interactions with the hosts, from apparently innocuous colonization to highly pathogenic invasion, emphasizes the need to better understand the host response to *Staphylococci*. Despite the huge health issues related to *S. aureus*, it is not yet known which immune mechanisms control the susceptibility of an organism to this kind of bacteria.

To date, most efforts have been focused on advancing knowledge on the pathogen and its remarkable repertoire of virulence factors. Less work has been carried out on host predisposition to *S. aureus* infections [6]–[8]. In humans, few studies have focused on the comparison of the transcriptional signatures of peripheral blood mononuclear cells from patients with *S. aureus* infections with those of healthy individuals [9]. Nevertheless, *S. aureus* infection has been examined in mouse strains that show varying degrees of resistance to infection, and innate immune mechanisms were thought to be linked with genetic control [10]. Several studies have been conducted on zebrafish, and phagocyte-dependent resistance was associated with a favorable outcome. Moreover, *Caenorhabditis elegans* and drosophila have also been exploited as invertebrate models to decipher the mechanisms of response to *S. aureus* [11]–[13]. The necessarily retrospective design of studies in humans means that it is particularly difficult to use them to identify immune mechanisms associated with protection against *S. aureus*. Moreover, in the other interaction models listed above, poor genetic variability limits their usefulness to study the genetic control of immune responsiveness.

Therefore, in the present study we have designed and implemented a new outbred animal model to explore the immune response of mammals to *S. aureus* infections. For this purpose, we used sheep from a divergent selection based on the susceptibility to staphylococcal infections of the mammary gland, a spontaneous and frequent infectious disease in this species [14]. Our initial experiments conducted on this animal resource clearly indicated that the two genetic lines showed a varying degree of natural resistance towards mammary infections and responded differently to staphylococcal infections [14]. The initial characterization of these animals supported the hypothesis that the immune outcome of such infections was different in the two lines, and therefore that this trait is probably under genetic control. Previous studies also suggest the involvement of genetic factors in the response to mammary infections [15].

It is generally considered that the mechanisms underlying resistance or susceptibility traits, which by essence are multifactorial, to staphylococcal infections are complex and therefore difficult to understand. A comprehensive study of the trait in *in vivo* infections seemed unwise at this stage due to the complexity and dynamic aspects of interactions and mediation in immune cells. Therefore, as a first step to identify what underlies the differences of predisposition to staphylococcal infection, we conducted an *in vitro* study of the interactions between dendritic cells (DCs) and *Staphylococcus*. DCs were our first choice due to their corner stone position between innate and adaptive immune responses [16]. Moreover, they share a large gene repertoire in common with other cell types such as macrophages, due to their myeloid precursor origin [17]. Previous studies have shown that DCs contribute to the various degrees of susceptibility towards several bacterial and parasitic pathogens, and their response on pathogen contact is of highly variable nature [18]–[21]. Hence, we hypothesized that DCs from animals showing different degrees of susceptibility to staphylococcal infections may have distinct transcriptional profiles upon infection.

Gene expression profiling has recently found applications in diagnosis, prognosis and exploring host-pathogen interactions in infectious diseases [9], [22]. Similarly, the identification of gene expression signatures indicative of disease subtypes through microarray technology has improved our understanding of the molecular basis of disease [23]. Moreover, transcriptome analysis has already been successfully applied to DCs, and provided new insights into immune responses [22], [24].

Therefore, we used gene expression profiling with an ovine-specific 15 k Agilent microarray to identify transcriptomic differences in ovine DCs from susceptible and resistant animals, in relation to staphylococcal immunity. Here, we show that the comparison of DC gene expression between both animal groups reveals striking differences and provides a basis for possible mechanisms involved in immune protection against *Staphylococci*.

## Results

### Characterization of ovine bone marrow-derived dendritic cell response to heat-killed *Staphylococcus aureus*

**Figure 1:**
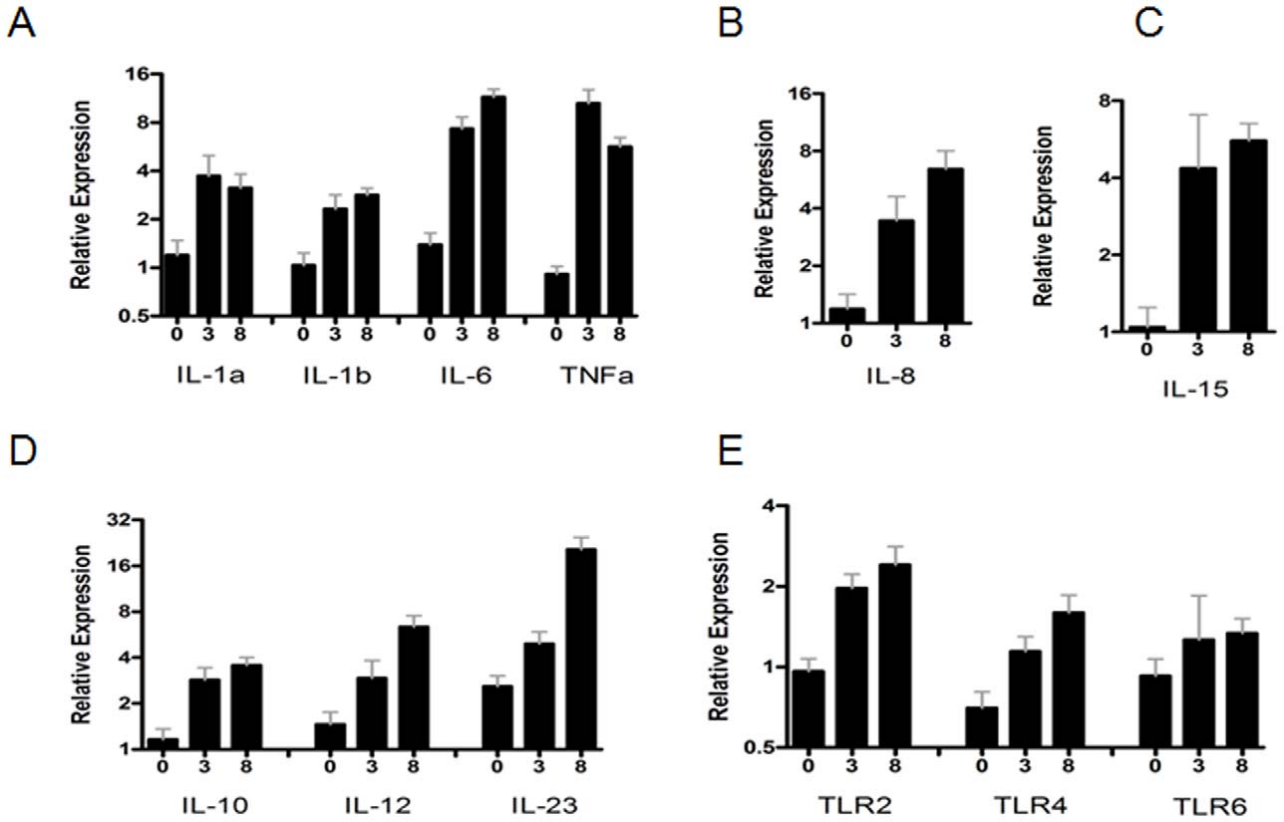
Pre-microarray validation of DC activation by RT-qPCR. Relative expression of mRNAs of different cytokines at three time points (0, 3 and 8 hrs post-stimulation) normalized by common reference genes (*rp19*, *hprt*, *tyr* and *gapdh*) by using 2^−ΔΔ^method. Data was combined from both resistant and susceptible animals in this experiment (A) Pro inflammatory cytokines (*Il1a*, −*1b*, −*6* and *tnfa*). (B) il8 (C) *il15* (D) T helper polarizing cytokines (*il10*, −*12* and −*23p19*) (E) Toll like receptors (*tlr2*, −*4* and −*6*).

In order to explore the response of dendritic cells from resistant and susceptible animals, we generated bone marrow-derived dendritic cells (BM-DCs) from sheep with known susceptibility according to their line and to results obtained from a previous infectious challenge (data not shown). After 6–7 days of culture, BM-DCs purity (>90%) and phenotype were assessed by flow cytometry. No difference was observed at this stage with available markers (data not shown). BM-DCs were stimulated with a commercial preparation (Pansorbin, Calbiochem) of heat-killed *S. aureus* (*HK-Sa*; 20 µg/ml) for 3 and 8 hrs. Following stimulation of BM-DCs, no differences were observed between the cells from both lines and their activation status as determined by flow cytometry was very similar. Based on data accumulated on DCs biology, the expression of a number of cytokine genes was quantified after 3 and 8 hrs of incubation (Fig. 1A). The transcripts of the pro-inflammatory cytokines *il1a*, *-1b*, *-6* and *tnfα* were considerably up-regulated 3 hrs post-stimulation, and high levels were maintained for all these cytokines after 8 hrs. *Il8* and *il15*, potent recruiters and activators of neutrophil granulocytes and NK cells respectively, were also highly expressed. Among T helper-polarizing cytokines, *il10*, *-12* and *-23,* mRNA expression was up-regulated (Fig. 1D). Interestingly, the transcription of the *il23* coding gene continued to increase as late as 8 hrs post-stimulation as compared to *il10* and *il12*. The expression of several Toll-like receptors (TLRs) was also measured, and a 2-fold increase in the transcription of *tlr2* and *tlr4* was observed, whereas the expression of *tlr6* showed little change and did not change much after stimulation.

Besides validating our *in vitro* assay and cell activation conditions, these data indicate that ovine BM-DCs are highly responsive to *HK-Sa*, since it induced the expression of a set of pro-inflammatory cytokine genes.

### Transcriptional analysis of DCs upon *HK-Sa* stimulation through microarray profiling

To further examine DC gene expression upon *HK-Sa* stimulation, all three experimental conditions described above (time points: 0, 3 and 8 hrs) were compared in a dye-swap experimental design.

Firstly, probe levels from the non-stimulated time-point (0 hr) and after stimulation with *HK-Sa* (3 and 8 hrs) were compared. Globally, ANOVA of microarray data revealed that a large number of genes were mobilized after *HK-Sa* stimulation (>3,000, with a corrected Benjamini-Hochberg (BH) false discovery rate p-value inferior to 0.01).

**Figure 2:**
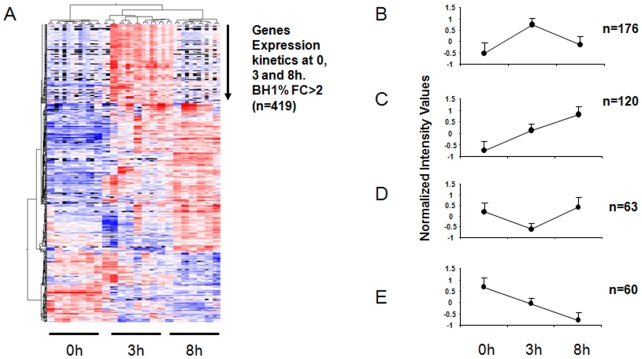
DC gene signature upon *Staphylococcus aureus* interaction at three different time points (0, 3 and 8 hrs). (A) Clustering analysis of differentially expressed genes (BH1%, FC>2, n = 419) at 0, 3 and 8 hrs post stimulation. (B) Rapidly exhausted genes subset (n = 176) (C) Post-stimulation up-regulated genes subset (n = 120) (D) Down-regulated genes at 3 hrs but up-regulated at 8 hrs post-stimulation (n = 63) (E) Post stimulation down-regulated genes (n = 60).

When considering only genes showing at least a 2-fold difference in expression at one of the three conditions (T3 vs T0, T8 vs T0 and T8 vs T3), a total number of 419 genes (Table S1) were found. Unsupervised hierarchical clustering of these genes indicated that they could be grouped into at least 4 subsets based on their mode of expression after stimulation (Fig. 2). Expression changes peaked at 3 hrs post-stimulation as compared to 8 hrs. Moreover, the number of up-regulated genes was higher than the number of down-regulated genes in the kinetics. The expression of most of the genes (n = 176) peaked at the 3-hr time-point and then decreased. The expression of 120 genes was increased at both post-stimulation time-points *i.e* 3 and 8 hrs, whereas 60 genes were down-regulated at these same time-points. The expression of a few genes (n = 63) tended to increase later on at 8 hrs. Fold changes (FC) for the differences in gene expression when comparing the three time-points (T3 vs. T0, T8 vs. T0 and T8 vs. T3) ranged from 9 to −7, 15 to −10 and 4 to −3, respectively. A list of the most differentially up- and down-regulated genes observed in the kinetics experiment is presented in the Table 1.

**Table 1:** List of the top differentially expressed genes (BH1% and FC>2) at 8 hrs post stimulation.

Gene regulation^a^	Gene symbol	Gene description	Probe ID	Genebank accession number
**Up** b	CYR61	Cysteine-rich, angiogenic inducer, 61	A_70_P036181	DY511533
	GMCSF	Colony stimulating factor 2 (granulocyte-macrophage)	A_70_P039321	NM_001009805
	TGLN	Transgelin	A_70_P069051	DY507201
	GEM	GTP binding protein over expressed in skeletal muscle	A_70_P063336	EE783131
	TNFRSF18	Tumor necrosis factor receptor superfamily, member 18	A_70_P007606	EE814846
	CARPB2	Cellular retinoic acid binding protein 2	A_70_P018436	DY495812
	EDN1	Endothelin 1	A_70_P051286	NM_001009810
	CSF3	Colony stimulating factor 3 (granulocyte)	A_70_P016862	LO7939
	DLL4	Delta-like 4 (Drosophila)	A_70_P059611	DY504356
	GADD45B	Growth arrest and DNA-damage-inducible, beta	A_70_P009501	DY491466
**Down** c	C-FOS	FBJ murine osteosarcoma viral oncogene homolog	A_70_P016316	EE779059
	CCR3	Chemokine (C-C motif) receptor 3	A_70_P051117	NM_001009241
	SLC38A9	Solute carrier family 38, member 9	A_70_P000886	FE026967
	IFI44	Interferon-induced protein 44	A_70_P006501	EE782051
	EDNRB	Endothelin receptor type B	A_70_P041851	AF349439
	LPAR6	Lysophosphatidic acid receptor 6	A_70_P039016	EE833955
	ARHGDIB	Rho GDP dissociation inhibitor (GDI) beta	A_70_P013521	EE872563
	TRAF3IP3	TRAF3 interacting protein 3	A_70_P054601	EE746178
	SULF2	Sulfatase 2	A_70_P049492	FE023233
	CDH1	Cadherin 1, type 1, E-cadherin (epithelial)	A_70_P016256	EE753172

a mode of expression of gene.

b up-regulation at three time point (0, 3, and 8 h).

c down-regulation at three time points (0, 3, and 8 h).

Globally, stimulation with *HK-Sa* modulated the gene expression of DCs and a number of genes involved in the pro-inflammatory response were over-expressed such as *il1a*, −*1b*, −*6* and *tnfα,* as previously indicated by the pre-microarray analysis by RT-qPCR. *Nfkb* was highly expressed at 3 hrs and as late as 8 hrs post-stimulation. *Cd40* and *cd83*, important markers of dendritic cell maturation were also up-regulated upon stimulation. Colony stimulating factors (*csf1*, *−2* and *−3*) involved in the production, differentiation and function of granulocytes and macrophages were among the most strongly up-regulated genes after interaction with *HK-Sa.* The expression of a few chemokines (*cxcl10* and *cxcl16*) and chemokine receptors (*cxcr5* and *ccr7*) was also up-regulated. On the other hand, staphylococcal exposure of dendritic cells also negatively modulated the expression of various genes, such as *adora3*, *ccr3*, *cfos*, *cdh1* and *sulf2* which all showed lower expression 3 and 8 hrs post-stimulation.

In order to validate our microarray results, the expression of 18 differentially-expressed genes (FDR p<0.01; FC>2) was measured using RT-qPCR. Microarray and RT-qPCR results are compared in table 2, which also shows a Pearson coefficient correlation of 0.9 between both methods (except for *il-15* that was found to be hugely up-regulated by RT-qPCR (54 fold) and much less so with the microarray (3.27 fold). Statistical Mann-Whitney tests were conducted on expression data and a minimum significance of p<0.05 was observed for all quantified genes.

**Table 2:** Post stimulation (3 and 8 hrs) differential kinetics of gene expression in BM-DCs, a comparison of fold change measured by microarrays and RT-qPCR.

	Microarray		RT-qPCR	
Gene Name	T(3 hrs)	T(8 hrs)	T(3 hrs)	T(8 hrs)
GCSF	3.6	9.9	5.43	11.14
MCSF	5.82	5.65	10.56	5.06
GMCSF	8.12	15.11	7.24	21.13
IL-6	3.37	2.91	5.25	4.94
IL-10	2.67	3.01	2.2	3.61
IL-12	1.4	4.54	1.76	5.23
IL-15	1.15	3.27	2.96	54.96
CASP3	−1.49	2.15	1.05	2.5
TNFRSF18	4.31	7.98	8.86	10.97
FGF18	6.78	6.5	7.52	6.35
CFOS	−6.5	−9.5	0.15	0.17
CCR3	−2.23	−3.58	0.51	0.34
EDNRB	−1.8	−4.1	0.35	0.17
SULF2	−1.2	−3.2	0.85	0.36
CDH1	−1.65	−4.92	0.37	0.17
MLLT1	5.09	−1	3.05	1.01
SFRS5	−4.2	−1.6	0.2	0.25
MTIE	−3.57	−2.98	0.96	0.87

RT-qPCR data are normalized according to multiple housekeeping genes (*rp19*,*hprt*, *tyr* and *gapdh*) by using 2^−ΔΔ^method and present expression ratio of resistant over susceptible animals.

In conclusion, ovine BM-DCs responded strongly to the stimulus and present a novel transcriptional profile upon stimulation with *S. aureus*. The scale of the gene mobilization observed 3 and 8 hrs post-stimulation revealed that gene reprogramming of dendritic cells occurs after exposure to *HK-Sa.* The gene profiles obtained are mostly associated with pro-inflammatory conditions.

### Comparison of transcriptional profiles of DCs from susceptible and resistant animals

**Figure 3:**
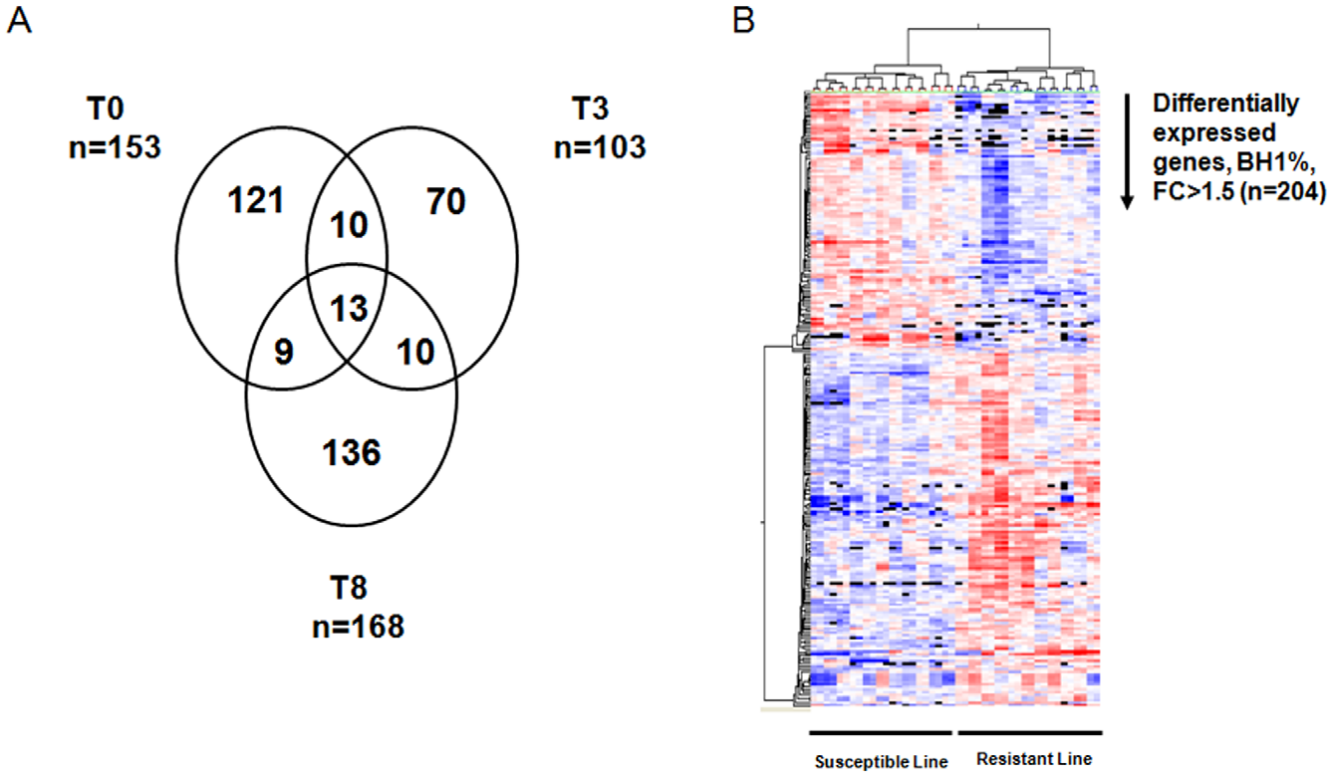
Transcriptional profiling of DCs in resistant and susceptible lines. (A) Venn diagram presenting number of differentially-expressed genes between the two lines at three different time points (Volcano plot analysis, FC>1.5). (B) Distinct transcriptional profiles of dendritic cells belonging to resistant and susceptible lines. Clustering analysis of differentially-expressed genes (ANOVA analysis, BH1%, FC>1.5, n = 204).

Using the same ANOVA, the gene expression between genetic lines was compared. Statistical analysis showed that 204 genes were differentially-expressed between DCs of the two lines (BH 1%, FC>1.5) in at least one of the three time points (Table S2). Overall, a FC>−2 to 3 in the expression of differentially-expressed genes was observed, with some quite important differences in expression (several genes showed FCs of −7 or +8). Moreover, animals belonging to susceptible and resistant lines could be grouped perfectly on the basis of this differentially-expressed gene list using Pearson-centered unsupervised hierarchical clustering (Fig. 3B). Furthermore, independent analysis using a volcano plot (p<0.01; FC>1.5) was performed to identify which genes were differentially expressed between the lines at each of the three time points (Table S3). Indeed, 153 genes were found to be differentially-expressed between both lines at T0. Among these differentially-expressed genes at T0, 72 and 81 genes were expressed at a higher level in the resistant and the susceptible line, respectively. Moreover, 103 genes (respectively 44 and 59 genes in resistant and susceptible animals) were modulated at T3, whereas 168 (respectively 95 and 73 in resistant and susceptible lines) genes were found to show different levels of expression at T8. Interestingly, only 13 genes were found in all three lists (Fig. 3A). Although the majority of the genes identified with this statistical method are on the ANOVA list, it provides additional information about differentially-expressed genes at each time point instead of variations in gene expression between the lines over the 8-hr time interval.

Among the ANOVA list of differentially-expressed genes, 106 genes showed higher expression levels in the resistant line, whereas 96 genes showed higher expression levels in the susceptible line. Table 3 presents a list of the most differentially-expressed genes between the lines at 8 hrs. Two genes *(Irf4* and *hook1)* showed a complex expression pattern over time and their expression varied in both cell lines at both 3 and 8 hrs post-stimulation. Among the differentially-expressed genes, a proportion of genes are known to have immune system-related functions. Expression of some other important immune genes (*ilrap, myd88*, *ido1*, *fabp7*, and *il12rb1*) was also found to be different between animals of the two lines. Genes coding for proteins involved in the classical complement pathway were also found to be differentially expressed between the lines upon *HK-Sa* stimulation (for example, *c1qa*, *b* and *c* were expressed at higher levels in the resistant line). Furthermore, expression of *cd59*, which is a regulator of the complement, was also found to be up-regulated at T8 in the resistant line. Expression of MHC 1 was higher in the resistant animals as compared to susceptible animals.

**Table 3:** List of the top differentially expressed genes (BH1% and FC>1.5) at 8 hrs post-stimulation between the resistant and susceptible lines.

Gene regulation^a^	Gene symbol	Gene description	Probe ID	Genebank accession number
**Up** b	SSRP1	Structure specific recognition protein 1	A_70_P054656	EE764907
	PRG3	Proteoglycan 3	A_70_P055056	EE768552
	MMP12	Matrix metallopeptidase 12 (macrophage elastase)	A_70_P006416	EE782850
	APRT	Adenine phosphoribosyl transferase	A_70_P027731	EE764223
	LAMA3	Laminin, alpha 3	A_70_P019151	EE782590
	GJB2	Gap junction protein, beta 2	A_70_P006901	EE757451
	CLDN4	Claudin 4	A_70_P059323	CD286830
	CLDN5	Claudin 5	A_70_P013486	DY498156
	UQCRQ	Low molecular mass ubiquinone-binding protein	A_70_P019896	EE823241
	TRIM2	Tripartite motif-containing 2	A_70_P066136	EE847713
**Down** c	POSTN	Periostin, osteoblast specific factor	A_70_P033761	EE857251
	TMEM87B	Transmembrane protein 87B	A_70_P057056	EE780570
	GPNMB	Glycoprotein (transmembrane) nmb	A_70_P017881	FE033937
	ADORA3	Adenosine A3 receptor	A_70_P051241	NM_001009775
	PIK3IP1	Phosphoinositide-3-kinase interacting protein 1	A_70_P038251	EE828789
	OLFML3	Olfactomedin-like 3	A_70_P059006	CU655165
	NAGA	N-acetylgalactosaminidase, alpha	A_70_P069481	EE822585
	FAM198B	Family with sequence similarity 198, member B	A_70_P009511	EE772814
	TNC	Tenascin C	A_70_P018186	EE825947
	TXNIP	Thioredoxin interacting protein	A_70_P016181	EE852061

a mode of expression of the gene.

b up-regulation at three time points (0, 3 and 8 hrs).

c down-regulation at three time points (0, 3 and 8 hrs).

Genes belonging and related to the TRAF family (*traf4*, *traf7*, and *traf3ip2*) were found to be differentially expressed between the lines, as were a few genes coding for matrix metalloproteinase family members (*mmp9* and *mmp12*). Among differentially-expressed genes, a number of genes belonged to the tripartite motif family (*trim2*, *trim39* and *trim45*). Several molecules involved in cell to cell adhesion were also differentially expressed between the lines, such as *cdh3* which was expressed at a higher level in the resistant animals, and *icam1* and *icam3* which were down-regulated in the same line. *Ssrp1, prg3, lama3,* and *gjb2* were among the most over-expressed genes in the resistant line whereas *postn*, *tmem87b*, *olfml3*, *naga* and *tnc* were the least expressed genes in that line.

**Figure 4:**
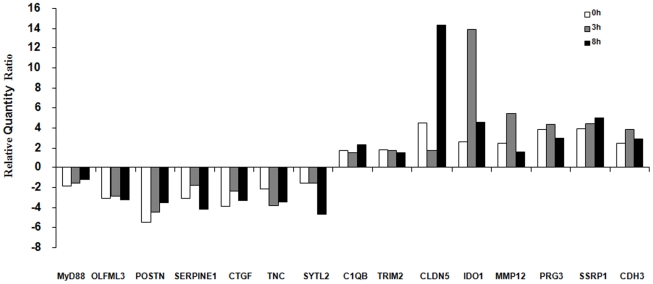
RT-qPCR analysis (Biomark system, Fluidigm) of differentially-expressed genes between resistant and susceptible lines. Data represent the expression ratio of resistant animals over susceptible animals at three different time points (0, 3 and 8 hrs). Relative data were normalized with common reference genes (*rp19*,*hprt*,*tyr* and *gapdh*) by using 2^−ΔΔ^method.

Fifteen differentially-expressed genes were selected and their quantification was done through RT-qPCR. For most of differentially-expressed genes between lines, a strong correlation (r = 0.7–0.9) existed between microarray and RT-qPCR results. Statistical Mann-Whitney tests were conducted on expression data and a minimum significance of p<0.05 was observed, validating at the same time the microarray results on a panel of genes. Data are presented as a ratio of genes of the resistant over the susceptible line (Fig. 4).

### Gene Network Analysis of differentially-expressed gene lists

Systemic identification and grouping of differentially-expressed genes into biological networks was performed using three data mining software packages (Ingenuity Pathway Analysis, Database for Annotation, Visualization and Integrated Discovery and Innate immune DataBase). Ingenuity Pathway Analysis (IPA) was conducted using human ortholog names (HUGO terms).

**Figure 5:**
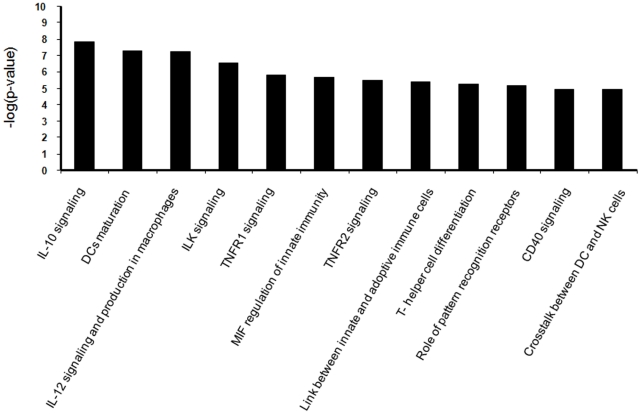
Top canonical pathways obtained by IPA analysis of differentially-expressed genes (n = 419, FC>2, BH 2%) between three time points (0, 3 and 8 hrs).

We first analyzed the networking of genes showing time-dependent expression after staphylococcal stimulation. Immunological disease and inflammatory processes were among the top biological functions that came up with highly significant p-values of (5.25×10^−16^–2.18×10^−06^) and (9.11×10^−16^–1.94×10^−06^) respectively. Moreover, 112 and 105 molecules belong to these networks and most of the genes from the initial 419-gene list were found in link with immune system pathways. IPA also gave a list of top canonical pathways such as IL-10 signaling, dendritic cell maturation, IL-12 signaling and production in macrophages, ILK signaling, TNFR1 signaling, MIF regulation of innate immunity, communication between innate and adaptive immune cells, T helper cell differentiation and the role of pattern recognition receptor in the recognition of bacteria and viruses with a FDR 1%, and a gene ratio>10% (Fig.5).

Moreover, biological roles were suggested for a list of the most-differentially expressed genes between the two lines (FDR 1%; FC>2, n = 112 genes). The most relevant biological networks were related to antimicrobial response, inflammatory response and infectious disease. Top canonical pathways included (1) acute phase response signaling, (2) leukocyte extravasation signaling and (3) complement system with significant p-values of 1.11×10^−02^, 1.54×10^−02^ and 1.33×10^−02^ respectively.

Further analysis of gene interactions and pathways was performed using the differentially-expressed genes at the three time points independently. We observed that top networks obtained at T0 deal with cell to cell signaling and interaction, inflammatory response and tissue development with 45 and 26 molecules taking part in these networks, respectively. Top diseases and disorders associated were inflammatory response, inflammatory diseases and immunological disease with highly significant p-values of (9.97×10^−07^–1.57×10^−02^), (5.46×10^−05^–1.57×^−02^) and (1.07×10^−04^–1.57×^−02^), respectively. Interestingly, the canonical pathways identified at T0 were IL-17 signaling, integrin signaling and B cell receptor signaling with statistical significance levels of 2.73×10^−03^, 4.19×10^−03^ and 5.57×10^−03^ respectively. Similarly, the comparison of gene expression between lines at the T3 time-point showed that (1) cell-to-cell signaling and interaction, cellular assembly and organization and (2) antimicrobial response, inflammatory response, cellular development are the main networks with 25 and 17 genes taking part in these networks, respectively. The main canonical pathway identified at T3 was ILK signaling (4.48×10^−03^). Moreover, independent IPA analysis of the T8 list indicated the involvement of complement system pathways and aryl hydrocarbon receptor (*ahr*) signaling pathway. The combined analysis of all three differentially-expressed gene lists (T0, T3 and T8) showed similar results to those obtained with ANOVA, but an additional canonical pathway dealing with the role of pattern recognition receptors in the recognition of bacteria and viruses showed up.

The biological significance of genes was also investigated by analyzing the over-expressed genes in each line separately at each time point by using DAVID (Database for Annotation, Visualization and Integrated Discovery). Major GO biological processes (BP) terms for susceptible animals were: (1) cell adhesion, cytoskeleton protein binding and (2) cell motion at T0, (1) glycosylation site N (2) signal peptide and (1) response to wounding at T3, and (1) endoplasmic reticulum part, (2) cell fraction, and (3) vesicle-mediated transport at T8.

**Figure 6:**
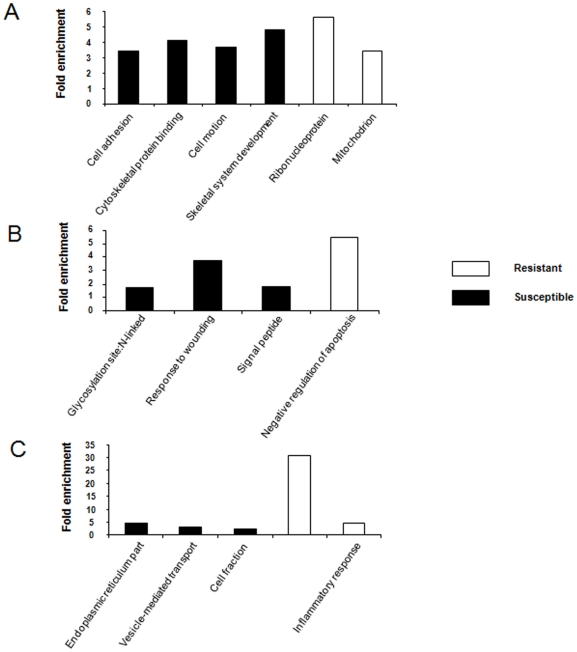
Enriched BP GO terms for over-expressed genes in each line at three independent time points by DAVID. (A) GO BP terms obtained at T0 (B) GO BP terms obtained at T3 (C) GO BP terms obtained at T8.

In the resistant line, the major GO BP terms obtained were (1) ribonucleoprotein and (2) mitochondrion at T0, no statistically significant GO BP terms were identified at T3, and (1) classical complement pathway and (2) response to wounding and inflammatory response were found to be significant GO BP terms at T8 (Fig. 6).

To identify more immune-related gene functions and interactions, a combined list of DE genes at T0, T3 and T8 was analyzed using InnateDB and transendothelial leukocyte migration was found to be the most significant GO term for susceptible animals when compared to resistant animals. The classical complement pathway was identified as the major pathway and is consistent with analysis using IPA and DAVID.

On the basis of differentially-expressed genes and biological processes obtained through networking analysis, it seems obvious that dendritic cells of animals belonging to resistant and susceptible lines have different transcriptional profiles not only at their differentiation steady state, but also upon interaction with *Staphylococcus*. Based on differentially-expressed gene lists, a certain number of hypotheses can be generated and linked with functions of the immune system. Furthermore, we observed that differentially-expressed genes belonged to both innate and adaptive immune response of DCs. Most of the genes identified showed a sustained up- or down-regulation and this clearly implies that the biological processes they sustain are important throughout the stimulation process. These differentially-expressed genes might explain the different outcomes of infections between lines obtained by divergent selection based on resistance to *Staphylococcus* infections.

## Discussion

We applied transcriptome analysis to investigate global gene signatures in BM-DCs after exposure to heat-killed *S. aureus*, and as a hypothesis-generating tool to identify differentially-expressed genes and pathways between animals having different degrees of susceptibility to staphylococcal infections. Dendritic cells have always been a center point of discussion in the context of infections by various pathogens, and their interaction with different bacteria and viruses have already been studied in details in different organisms [25]–[30].

The dendritic cells used in our study were generated from bone-marrow progenitors in the presence of recombinant ovine GM-CSF. Generation of dendritic cells from bone- marrow cultures is considered a standard protocol to study interactions with pathogens and is widely used in different species [31]–[34]. Although DCs could be isolated from mammary tissue or collected from lymph ducts in sheep, we preferred *in vitro*-generated DCs to avoid the interference of environmental (nutritional, hormonal, …) factors on DC gene profiles. In addition, the presence of pre-existing infections within such tissue could interfere with the analysis of basal gene expression, and jeopardize the study of the genetic control of the response to *Sa*.

The analysis of microarray-based transcriptional data from DCs were conducted to test two major axis of our experiment, *i.e.* gene modulation after exposure to *HK-Sa* over a time scale of 8 hrs, and comparison of the differential transcriptomic profiles between two divergent lines upon contact with *Sa*. The greater number of genes expressed at 3 hrs clearly indicates a rapid response of ovine DCs to staphylococcal stimulation.

The expression of major pro-inflammatory cytokines and chemokines (*il8* and *cxcl10*) was up-regulated at 3 and 8 hrs as previously described by others [35]. This clearly shows that stimulated dendritic cells are able to attract immature DCs, and activate T cells as well as other immune cells of the innate immune system at the site of inflammation. The genes coding for colony-stimulating factor (*csf1*, *−2* and –*3*) and *il15,* respectively involved in the production, differentiation and function of granulocytes, macrophages and NK cells, were highly up-regulated after DC stimulation. Over-expression of conventional and T helper response cytokines shows the potency of stimulated DCs to prime and induce expression of Th1-, Th2- and Th17-related cytokines by CD4+ T cells, and regulate the inflammatory response associated to that kind of infection. More precisely, the higher expression levels observed for *il6* and *il23* strongly suggest the involvement of Th17 response to *Staphylococcus* infections as recently described [36], [37], although no difference between the lines has yet been identified for that particular T-cell phenotype.

Gene network analyses show that the IL-10 signaling pathway is mobilized after stimulation with *HK-Sa*. After interaction with gram-negative bacteria, DCs express high levels of IL-12 and produce no or very little IL-10 [38]. In contrast, *HK-Sa-*stimulated DCs equally transcribe *il10* and *il12* as early as 3 hrs after stimulation, although expression of *il12* tends to be higher at 8 hrs post-stimulation. This could explain why staphylococcal infections often become chronic due to the immunosuppressive activity of IL-10, as compared to infections by gram-negative bacteria where inflammation is more acute and of higher magnitude. Among other mobilized genes, we found that transcription of *Irak2,* which plays a central role in the TLR-NFkappaB signaling axis, was increased. If it is then translated into protein, its up-regulation could create an amplification loop [39]. Conversely, *cfos* which is involved in the same signaling pathway was repressed.

A total of 73 genes were down-regulated after interaction of dendritic cells with *HK-Sa*. Among these down-regulated genes, the chemokine receptor *ccr3,* which is highly expressed in immature dendritic cells, and reported to be repressed in inflammatory conditions was found to be down-regulated [40]. By contrast, the transcription of markers of DC maturation (*cd40* and *cd83*) was increased after stimulation and clearly showed that maturation has occurred, whereas genes involved in cell to cell signaling and cell adhesion (*sulf2* and *cdh1*) were repressed.

Thus, our results and list of genes agree with some of the previously published work on DCs in inflammatory conditions [22], [41]–[44]. Indeed, Torri *et al.* identified a set of 54 genes associated with inflammation and among these genes, 14 genes (*ccl22*, *ccr10*, *ccr7*, *cd40*, *cd83*, *eif2b1*, *gadd45b*, *il4l1*, *irak2*, *il-6*, *il-1b*, *il12rb1*, *il-2ra* and *tnfα*) are present in our gene list [22]. This indicates that a similar pattern of gene expression exists between species in inflammatory conditions.

In a second step, we concluded that dendritic cells from two different genetic groups present distinct transcriptional profiles and that this could explain their varying ability to overcome *S. aureus* infection. A total of 204 genes were found to show different expression profiles in BM-DCs from the two lines over a time scale of 8 hrs. Interestingly, gene expression was already different before stimulation. We may therefore speculate that these differences actually reflect the genetic origin of DCs or that they are perhaps related to the GM-CSF-driven differentiation of DCs and a different capacity of the two lines to respond to GM-CSF. Indeed, in mice, two lines have been generated on the difference of intensity of the acute inflammatory response (AIR). BM precursor cells from AIRmax mice have been reported to display higher levels of expression of CD131, the beta chain of GM-CSFR, at their cell surface [45]. Using gene networking analysis, we found that two genes (*rras2* and *bcl2a1*) involved in GM-CSF signaling are up-regulated in the resistant line. Further investigations are needed to clarify this observation in our model.

The production of inflammatory and T helper-differentiating cytokines by DCs has been shown to contribute to severity of bacterial infections [46], [47]. In the past, Th2 type cytokines IL-4 and IL-10 were associated with resistance to *Staphylococcus* infection in mice [47]. Previous studies have shown that DCs from *L. major* susceptible mice produced low quantities of IL-1α and IFNγ in response to LPS and this was thought to be responsible for different outcomes after infection by the parasite [21]. By contrast, no significant difference was observed in the expression of any major immune system pillar cytokines and chemokines between susceptible and resistant animals in our study.

Networking analysis of differentially-expressed genes between two lines revealed two major pathways. In the susceptible line, genes involved in the leukocyte transendothelial migration were expressed at a higher level which could be related to our clinical findings since the selection criteria of the animals are based on elevated leukocyte concentration in the milk. In resistant animals, genes involved in the classical complement pathway were expressed at a higher level.

Among the differentially-expressed genes, few genes involved in the IL-1R signaling were over-expressed in the susceptible line. Expression of *myd88* and *ilrap* was lower in the resistant line. Miller *et al.* extensively described the role of the IL-1R/Myd88 and TLR2/Myd88 pathways in the recruitment of neutrophils and clearance of staphylococcal infections in mice, and demonstrated the dominant role of IL-1R signaling over TLR signaling [48]. They showed that the clearance of *Staphylococcus* infection in chimera mice reconstituted with IL-1R-deficient or Myd88-deficient BM was the same. Although the expression of IL-1R or MyD88 by recruited BM-derived cells had no impact on the outcome of the infection, IL-1R/Myd88 signaling by resident cells, at the site of infection, controlled the outcome of infection. Hence, it is difficult to conclude at this stage whether there is a difference in the signaling cascade from IL-1R/TLR between the two lines but the up-regulation of both *myd88* and *ilrap* genes certainly suggests that IL-1R signaling is increased in the susceptible line.

DCs are known to promote Th1 response via the secretion of IL-12. In our study the expression of *Il12rb1,* a chain of the IL-12 receptor complex, was increased in the cells of the resistant animals. Although the expression of *il12* was not significantly different between the lines, resistant animals tended to produce more *il12* mRNA after stimulation (1.6 fold). Since IFNγ is known to stabilize the expression of IL-12Rβ, it is tempting to speculate that the resistant line has a more potent Th1 response due to an increased production of IFNγ compared to the susceptible line [36]. Previous studies have shown that default of *Il12rb1* gene expression is associated with susceptibility towards Mycobacteria and malarial infections [49], [50], but until now, predisposition to staphylococcal infections has not been reported in relation with this default.

It is generally considered that not only initiation of an inflammatory response is required but also that complete shutdown of the inflammatory process is essential for the ultimate outcome of an infection. Interestingly, we observed that *ido1* (indoleamine 2, 3-dioxygenase 1) expression was increased in the resistant line. *ido1* is reported to help catalyzing tryptophan and to block the inflammatory response by promoting the differentiation of regulatory T cells (Treg) [51]. Some previous studies have shown that the inhibition of *ido1* promotes the production of NK cells and the production of IFNs and further aggravates inflammation [52]. Altogether, the high level of *ido1*expression in the resistant line may suggest that the DCs from resistant animals can not only implement an efficient inflammatory response against staphylococcal infections via conventional IL-1R/TLR pathway signaling, but can also shut it down as soon as infection clearance begins. This could help to prevent the detrimental effects observed in the susceptible line due to an uncontrolled and chronic inflammatory response.

During inflammation, the clearance of apoptotic bodies is thought to be an important physiological event to prevent the promotion of inflammation and autoimmunity whilst also limiting tissue toxicity. *C1q,* the first component of complement system, is one of the key molecules involved in the recognition and clearance of apoptotic bodies [53]–[55]. Interestingly, one of the gene networks obtained in our study contains all three subcomponents of *C1q*, which showed highly up-regulated expression levels in the resistant line. *C1q* is not only involved in the uptake of apoptotic cells by DCs, but also in phagocytosis, which installs a combination of IL-6 and TGFβ production through recognition of pathogen-associated molecular patterns [56] and phosphatidylserine [57] exposed on apoptotic cells. This combination of IL-6 and TGFβ instructs Th17 differentiation [58]. Furthermore, the ingestion of apoptotic cells can convert immature DCs into tolerogenic DCs and therefore ultimately leads to the conversion of naïve T cells into Foxp3-positive Treg [59]. Hence, the up-regulation of *C1q* in the resistant animals suggests that unnecessary inflammation might be avoided by the efficient clearance of apoptotic bodies, programming of the cytokine profile towards Th17 differentiation and simultaneous regulation of the inflammatory response through Treg.

Among the differentially-expressed genes between lines, several TRAF family genes, such as *traf4,* were over-expressed in the resistant line. *Traf4,* unlike other genes of TNF receptor associated family genes, is involved in DC migration. Reduced migration of dendritic cells was observed in *traf4*−/− mice [60]. Hence, DC migration could be faster in the resistant line. Moreover, several genes belonging to the matrix metalloproteinase family (*mmp1* and *mmp12*) are over-expressed in the resistant line. Although its antimicrobial properties have not been reported in dendritic cells, the role of MMP12 in macrophages has been recently identified. *Mmp12* ^−/−^ mice were shown to have impaired bacterial clearance and increased mortality upon infection with gram-positive bacteria [61]. Several genes belonging to two major families, the claudin family (*cldn1*, *cldn4* and *cldn5)* and the TRIM family (*trim2*, *39* and *45*), were found to be differentially modulated after stimulation in the two lines. Since the importance of the genes belonging to the claudin and Trim families has been evidenced in the context of viral infections and cancer, it is worth noting that they may also play a role in the response to bacterial infections.

In conclusion, we have successfully exploited transcriptomic analysis to obtain a *Staphylococcus-*induced ovine DC gene signature. We have also developed a series of hypotheses on the basis of the genes found to be differentially-expressed between animals of the susceptible and resistant lines. Whether or not the susceptibility towards *Staphylococcus* infections completely depends upon the differentially-expressed genes identified using DC transcriptomics remains unknown at this stage. But the differences observed for genes with immune-related functions lead us to speculate on a central role for dendritic cells in the context of staphylococcal infections. Hence, the differentially-expressed genes identified in this study should be further investigated using laboratory models to confirm our hypothesis. To our knowledge, this the first study investigating immunity against *S. aureus* in an outbred animal model of such genetic variability. Our study has opened some new avenues which will need further exploration and lead to a better understanding staphylococcal immunity and resistance.

## Materials and Methods

### Animals

Two-year old ewes (Lacaune breed) from resistant (n = 4) and susceptible lines (n = 4) were issued from a divergent selection on the basis of estimated breeding values for the somatic cell score, an indicator of intra-mammary infections; they had no parental links. Previous studies conducted on these genetic lines indicated a significant difference of susceptibility to intramammary infections by *Staphylococci* [14]. Since these infections are principally due to *Staphylococcus* spp, the genetic trait is highly related to the susceptibility to this bacteria genus. Moreover, the ewes used in that study were challenged twice with two different species (*Staphylococcus epidermidis* and *S. aureus*) and the results of bacteriology, disease severity and abscess formation indicated a significant difference of susceptibility between the groups (EF, RR, GF, manuscript in preparation). The ewes were thus chosen as representative of their genetic lines. Animals were sacrificed in accordance with local regulations (Agreement number N°31-2010-67). This study was approved by the INRA animal ethics committee (France).

### Generation and stimulation of bone marrow-derived dendritic cells

The DCs used in this study were generated from bone-marrow progenitors following a protocol previously described by our group [62]. Briefly, bone marrow was prepared from the sternum after longitudinal section in two halves and scraping of the red marrow. Bone marrow cells were released by crushing the marrow in a 100 mm Petri dish filled with 10 ml HBSS medium (Invitrogen). Cells were suspended by carefully pipetting up and down, and were then passed through a 100-µm nylon mesh to remove small pieces of bone and debris. Red blood cells were lysed by ACK treatment for 2–3 min at room temperature. Cells were then suspended in complete RPMI medium and cultured in 100 mm bacteriological dishes at 37°C, 5% CO_2_ for 7 days. Complete medium was RPMI 1640 (Invitrogen) containing 2 mM glutamine, 25 mM HEPES, 10% FCS, 1% penicillin/streptomycin, 1% sodium pyruvate, 1% essential amino acids, 50 µM β-mercaptoethanol. cRPMI medium was supplemented with 20 ng/ml ovine recombinant GM-CSF. Fresh medium containing the same concentration of recombinant cytokine was added every 3 days. After seven days of culture, cells were characterized by flow cytometry. BM-DCs were seeded in cell culture plates at a concentration of 1.5x10^6^ cell/ml and stimulated with 20 µg/ml Pansorbin for 3 and 8 hrs.

### RNA extraction, amplification and labeling

Total RNA was extracted using the classical extraction method with phenol/chloroform (TRIzol, Invitrogen). Extracted RNA was further purified on RNeasy columns (Qiagen). Quantification was performed using a spectrophotometer (NanoDrop Technologies) and RNA quality was assessed using an Agilent Bioanalyzer 2100 slide. A RNA Integrity Number (RIN) index above 9 was measured for all samples. 200 ng of RNA was converted into double-stranded cDNA and amplified with the Amino Allyl Message Amp II aRNA amplification procedure (Ambion kit) and then labelled with Cy3 and Cy5 for hybridization in a two-colour dye-swap experimental design.

### Hybridization, scanning and raw data storage

Three ovine 019921 slides (Agilent Technologies), with eight microarrays each containing 15,008 probes were used. The chips were hybridized with labeled RNA at 65°C for 17 hrs and then washed according to the manufacturer's protocol. Intensity values were collected with a 4000B Axon scanner. Two channel images were imported into the Agilent's Feature Extraction software for feature (spot) finding and alignment. Features were flagged as present/absent, saturated/unsaturated, uniform/non-uniform and pixel intensities for features and background were recorded.

Pearson correlation coefficients taken from plotting signal intensity values of dye-swap chips across all genes validated that duplicate experiments were nearly similar. Output Feature Extraction result (.txt) files were transferred into GeneSpring GX 11. LOESS-normalized data were imported as a single channel data to perform probe by probe analysis and a percentile shift to the median was performed for normalization across arrays. Data were filtered to remove spots that were of very low intensity, saturated, or not uniform. In order to keep only genes that were very representative of one condition, only genes that were positively flagged in all samples for at least one condition (resistant line at T0, T3, and T8, and susceptible line at T0, T3, and T8) were conserved for further analysis.

### Annotation of ovine microarray

On the ovine array, 15,208 different probes are present, but only 8,847 of these are annotated with the Human ortholog HUGO Gene Nomenclature Committee (HGNC) (http://www.sigenae.org/, sheep oligo annotation version 6 of 2010/06/14). More genes were annotated using GeneBank and Basic Local Alignment Search Tool program on the NCBI website (http://blast.ncbi.nlm.nih.gov/Blast.cgi). A threshold value of 80% hit score was considered as a minimum criterion.

### Statistical analysis of microarray data

Statistical analysis of microarray data was carried out using the commercially available software package Genespring®. Two-way ANOVA by Line and Time was performed to select genes that were differentially expressed between different conditions with a p-value corrected by Benjamini Hochberg false discovery rate. The interaction between Line and Time was tested, but it was not significant and was removed from the statistical model. The expression profiles for these genes were clustered over the line or over the time using the hierarchical clustering of GeneSpring based on the Pearson-centered gene distance. Information about this experiment has been deposited in NCBI Gene Expression Omnibus (GEO) with accession number (GSE24448). All data is Minimum Information About a Microarray Experiment (MIAME) compliant.

### Gene networking analysis

Three freely available software packages were used to interpret the biological functions of the gene lists. Initially networking analyses were conducted using Ingenuity Pathway Analysis, version 7.5 (http://www.Ingenuity.com). In addition to generating biological networks from a list of selected genes, this software provides biological functions and canonical pathways from HUGO ortholog names of the genes that were imported into the program. A network of genes is created when a gene regulates the function of another one. To generate a network, the IPA software adds other genes and/or different molecules that are linked with two focus genes. Gene Ontology (GO) Term analyses were performed with DAVID (Database for Annotation, Visualization and Integrated Discovery) (http://david.abcc.ncifcrf.gov/) by using an independent list of differentially-expressed genes at three time points (T0, T3 and T8) separately. Transcription factor binding sites (TFBS) over-represented in differentially-expressed gene list were identified using InnateDB (http://www.innatedb.com).

### Reverse transcription quantitative polymerase chain reaction (RT-qPCR)

cDNAs were generated from 300 ng of clean total RNA of 24 samples using the Superscript III First Strand Synthesis System Kit (Invitrogen), and random hexamer primers, following the manufacturer's instructions.

The relative mRNA expression levels were verified using the same RNA samples that had been analyzed in microarrays. For quantification of mRNA transcripts, primer pairs were designed using Primer3 [63] based on the relevant ovine mRNA sequences and synthesized commercially (Eurogentec). The specificity of designed primers was checked with other tools (BLAST, http://blast.ncbi.nlm.nih.gov/) and Primer Express̀. For some genes for which ovine sequence was not available, a comparative gene alignment of bovine, human, rat and mouse sequences was preformed, and primers were then designed on the most conserved regions between these species. Optimal annealing temperatures were determined for each primer pair, and the primers were checked for absence of primer dimers and efficiency before use. Primers used in RT-qPCR experiments are listed in Table S4.

RT-qPCR was performed following two different protocols according to the number of genes to be tested. Expression of few genes was tested in pre-microarray experiments and RT-qPCR reactions were carried out on a 7300 machine (Applied Biosystems). All assays were carried out in duplicate and each reaction contained 5 µl of diluted cDNA with 2.5 µl (0.5 µM) of each forward and reverse primer along with 12.5 µl of Power Syber Green PCR Master Mix (Applied Biosystems).

The high-throughput microfluidic qPCR platform BioMark™ (Fluidigm) was used for post-microarray qPCR analysis running the 48.48 dynamic-array. Pre-amplification of cDNA for all samples was done with a pool of forward and reverse primers. Five µl pre-amplification reaction contains 1.25 µl of pool of primers with 2.5 µl TaqMAN®PreAmp Master Mix and 1.3 µl cDNA. Pre amplification of cDNA was carried out on an ABI thermocycler with an initial step of activation (95°C for 10 min) followed by 14 cycles of two steps (95°C for 15 sec and 60°C for 4 min).

The sample reaction mixtures had a final volume of 6.7 µl and made of 1.7 µl of diluted pre-amplified cDNA and 5 µl of sample pre-mix containing 20x EvaGreen, 20x DNA Binding Dye Sample loading reagent and 2x Taqman Gene Expression Master mix. The primer reaction mixtures had also a final volume of 6.7 µl and were made up of 5 µl of 2X Assay Loading reagent (Fluidigm) and TE 1X and 1.7 µl of a mixture of reverse and forward primers (20 µM). Empty dynamic array was first primed with oil solution in the NanoFlex™ 4-IFC Controller (Fluidigm) to fill its control valves for 20 min.

Five µl of sample reaction mixtures were then loaded into the sample wells carefully avoiding any bubbles, and 5 µl of primer reaction mixtures were loaded into the assay wells. The dynamic array was then placed again into the NanoFlex™ 4-IFC Controller for loading and mixing. The mixing takes place by diffusion between the reaction chambers filled with sample reagent and adjacent containers filled with the appropriate primers mix for 90 min. Loaded dynamic array was then transferred to the BioMark™. The BioMark™ qPCR cycling program was 50°C for 2 min for amplification phase and 10 min at 95°C for activation of the hot-start enzyme, followed by 35 cycles of denaturation at 95°C for 15 s, annealing at 60°C for 1 min, and elongation at 72°C for 20 s. Melting curve analysis was performed after completion of the qPCR collecting fluorescence intensities between 60 and 95°C.

### RT-qPCR data analysis and normalization

Specific amplification of each targeted cDNA was confirmed by melt curve analysis. Measured Ct values were exported from the BioMark™ software to Excel for data analysis. qPCR technical replicates of samples were averaged and expression ratios were calculated by the delta delta Ct method normalized to the multiple housekeeping genes [64]. Statistical significance of analysis was calculated using Mann-Whitney tests. Beforehand the stability of 10 house keeping genes, previously mentioned in the literature, was checked in our 24 samples and data obtained was then analyzed by GeNorm, a software package freely available for research use. Hence, RT-qPCR data were normalized against the four most stable genes (*rp19*, *hprt*, *tyr* and *gapdh*) found by GeNorm analysis.

## Supporting Information

Table S1 **List of the differentially expressed genes in the time course identified by ANOVA of microarray data (FC>2, BH1%, n = 419) at 0, 3 and 8 hrs.**
(XLS)

Table S2 **List of the differentially-expressed genes between resistant and susceptible lines at 0, 3 and 8 hrs by ANOVA of microarray data (FC>1.5, BH1%).**
(XLS)

Table S3 **List of the differentially-expressed genes between the two lines by Volcano plot analysis (FC>1.5) at 0, 3 and 8 h independently.**
(XLS)

Table S4 **List of primer sequences used in RT-qPCR.**
(XLS)
